# Supplementary material for: Transcriptome Analysis of Light-Regulated Monoterpenes Biosynthesis in Leaves of Mentha canadensis L
Source: Plants (Basel). 2021 May 7;10(5):930. doi: 10.3390/plants10050930 (PMC8148558; doi:10.3390/plants10050930)
Supplement: Supplementary file 1 [file plants-10-00930-s001.zip › Supplementary Figure S4 revised.pdf]

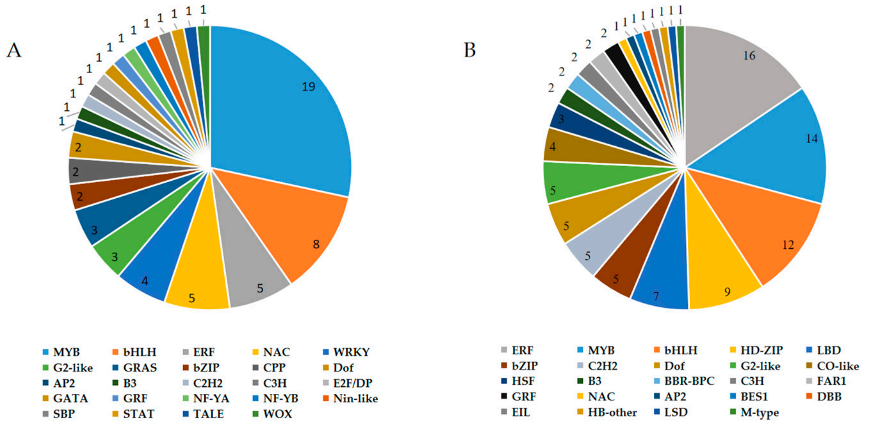

Supplementary Figure S4 Distribution of transcription factor (TF) families. A Distribution of 67 TFs have the similar expression pattern with terpenoids synthesis pathway genes. B Distribution of 67 TFs have the opposite expression pattern with terpenoids synthesis pathway genes which were down-regulated by darkness and up-regulated after recovery light.
