# Supplementary material for: Transcriptome Analysis of Light-Regulated Monoterpenes Biosynthesis in Leaves of Mentha canadensis L
Source: Plants (Basel). 2021 May 7;10(5):930. doi: 10.3390/plants10050930 (PMC8148558; doi:10.3390/plants10050930)
Supplement: Supplementary file 1 [file plants-10-00930-s001.zip › Supplementary of sequencing data.pdf]

Sequence data of mint (*Mentha Canadensis*) menthol biosynthesis pathway genes

>Seq1 [organism=Mentha canadensis] geranyl diphosphate synthase large subunit

ATGAGTGCTCTTGTTAATCCTGTGGCGAAATGGCCTCAGACGATCGGCATTAAAGATGTTCACGGCGGCCGG
AGGCGGAGATCCAGATCCACTCTCTTTCTCTCTCATCCACTTCGCACCGAAATGCCTTTCTCTCTCTACTTCTC
ATCCCCCCTCAAAGCTCCCGCCACTTTTTCCGTTTCTGCAGTTTATACCAAAGAAGGCAGCGAAATTAGGGA
TAAAGATCCGGCGCCTTCGACTTCGCCGGCGTTCGATTTCGACGGATACATGCTCCGGAAGGCGAAATCCGT
CAACAAGGCGTTGGAAGCGGCGGTGCAGATGAAGGAGCCGCTGAAGATCCACGAGTCCATGCGGTACTCC
CTTCTCGCCGGCGGCAAGAGAGTGCGTCCGATGCTGTGCATCGCGGCCTGCGAGCTCGTCGGCGGCGACG
AGTCCACGGCGATGCCGGCGGCCTGCGCCGTCGAGATGATCCACACGATGTCGCTGATGCACGACGACCTC
CCCTGCATGGACAACGACGACCTCCGCCGCGGCAAGCCGACGAACCACATGGCTTTCGGCGAGAGCGTGG
CGGTCCTCGCCGGCGACGCGCTCCTCTCCTTCGCGTTCGAGCACGTGGCAGCGGCGACCAAAGGCGCGCC
GCCGGAGCGGATCGTGAGGGTCCTCGGCGAGCTGGCGGTCTCGGTCGGGTCGGAGGGGCTGGTGGCGG
GGCAGGTGGTGGACGTCTGCTCGGAGGGGATGGCGGAGGTCGGGCTGGACCACCTCGAGTTCATCCACC
ACCACAAGACGGCGGCGCTGCTGCAGGGGTCGGTGGTTCTGGGGGCAATTTTGGGCGGCGGAAACGAGG
AGGAGGTGGCGAAGTTGAGGAAATTCGCGAATTGCATCGGATTGCTGTTTCAGGTGGTGGACGATATCCTA
GATGTGACGAAATCGTCCAAGGAATTGGGGAAGAAGGCGGGGAAGGATCTGGTGGCGGATAAAACCACA
TATCCGAAGCTAATAGGCGTGGAGAAATCCATGGAATTCGCGGATCGGTTGAACAGGGAGGCGCAGGAGC
AGCTCCTCCATTTTCATCCTCATAGGGCAGCTCCATTGATTGCTCTCGCCAATTATATTGCTTATAGGGACAATT
GA

>Seq2 [organism=Mentha canadensis] geranyl diphosphate synthase small subunit

ATGGCCATTAATCTCTCCCATATCAACTCCAAAACATGTTTCCCTCTCAAAACAAGATCTGATCTCAGCCGTTC
TTCTTCCGCGCGTTGTATGCCAACTGCCGCCGCTGCCGCCTTCCCCACTATCGCCACCGCCGCCCAAAGTCA
GCCGTACTGGGCCGCCATCGAGGCCGACATAGACAGATACCTGAAGAAATCCATCACAATAAGGCCGCCGG
AGACAGTTTTCGGGCCCATGCACCACCTCACCTTCGCCGCCCCAGCCACCGCCGCCTCCGCCCTATGCTTGG
CGGCGTGCGAGCTCGTCGGCGGCGACCGAAGCCAAGCCATGGCAGCCGCGGCGGCGATCCATCTCGTGCA
CGCGGCAGCCTACGTCCACGAGCACCTCCCTCTAACCGACGGGTCGAGGCCCGTATCCAAGCCCGCAATCC
AGCACAAGTACGGCCCGAACGTCGAGCTCCTCACCGGAGACGGGATTGTCCCGTTCGGGTTTGAGTTGCTG
GCCGGGTCAGTGGACCCGGCCCGAACAGACGACCCGGATAGGATTCTGAGAGTTATAATAGAGATCAGTCG
GGCCGGCGGGCCGGAGGGAATGATAAGCGGGCTGCATAGGGAAGAAGAAATTGTTGATGGAAATACGAG
TTTAGACTTCATTGAATATGTGTGCAAGAAAAAATACGGCGAGATGCATGCTTGCGGCGCGGCTTGTGGAGC
CATATTGGGCGGCGCAGCCGAGGAGGAGATTCAGAAGCTGAGGAATTTCGGGCTTTATCAAGGAACTCTC
AGAGGAATGATGGAAATGAAAAATTCTCATCAATTAATTGATGAGAATATAATTGGAAAATTGAAAGAATTG
GCTCTCGAGGAGTTGGGAGGCTTCCACGGGAAGAACGCTGAGCTGATGTCGAGCCTTGTAGCCGAGCCGA
GCCTTTACGCGGCTTAG

>Seq3 [organism=Mentha canadensis] limonene-3-hydroxylase

ATGGAGCTCCAGATTTCGTCGGCGATTATAATCCTTGTAGTAACCTACACCATATCCCTCCTAATAATCAAGCA
ATGGCGAAAACCGAAACCCCAAGAGAACCTGCCTCCGGGCCCGCCGAAGCTGCCGCTGATCGGGCACCTC
CACCTCCTATGGGGGAAGCTGCCGCAGCACGCGCTGGCCAGCGTGGCGAAGCAGTACGGCCCAGTGGCGC
ACGTGCAGCTCGGCGAGGTGTTCTCCGTCGTGCTCTCGTCCCGCGAGGCCACGAAGGAGGCGATGAAGCT
GGTGGACCCGGCCTGCGCGGACCGGTTCGAGAGCATCGGGACGAAGATCATGTGGTACGACAACGACGAC
ATCATCTTCAGCCCCTACAGCGTGCACTGGCGCCAGATGCGCAAGATCTGCGTCTCCGAGCTCCTCAGCGCC
CGCAACGTCCGCTCCTTCGGCTTCATCAGGCAGGACGAGGTGTCCCGCCTCCTCGGGCACCTCCGCTCCTC

GGCCGCGGCGGGGGAGGCCGTGGACCTCACGGAGCGGATAGCGACGCTGACGTGCTCCATCATCTGCAG
GGCGGCGTTCGGGAGCGTGATCAGGGACCACGAGGAGCTGGTGGAGCTGGTGAAGGACGCCCTCAGCAT
GGCGTCCGGGTTCGAGCTCGCCGACATGTTCCCCTCCTCCAAGCTCCTCAACTTGCTCTGCTGGAACAAGA
GCAAGCTGTGGAGGATGCGCCGCCGCGTCGACGCCATCCTCGAGGCCATCGTGGAGGAGCACAAGCTCAA
GAAGAGCGGCGAGTTTGGCGGCGAGGACATTATTGACGTACTCTTTAGGATGCAGAAGGATAGCCAGATCA
AAGTCCCCATCACCACCAACGCCATCAAAGCCTTCATCTTCGACACGTTCTCAGCGGGGACCGAGACATCAT
CAACCACCACCCTGTGGGTGATGGCGGAGCTGATGAGGAATCCGGAGGTGATGGCGAAAGCGCAGGCGG
AGGTGAGAGCGGCGCTGAAGGGGAAGACGGACTGGGACGTGGACGACGTGCAGGAGCTTAAGTACATG
AAATCGGTGGTGAAGGAGACGATGAGGATGCACCCTCCGATCCCGTTGATCCCGAGATCATGCAGAGAAGA
ATGCGAGGTCAACGGGTACACGATTCCGAATAAGGCCAGAATCATGATCAACGTGTGGTCCATGGGTAGGA
ATCCTCTCTACTGGGAAAAACCCGAGACCTTTTGGCCCGAAAGGTTTGACCAAGTCTCGAGGGATTTCATG
GGAAACGATTTCGAGTTCATCCCGTTTGGAGCTGGAAGAAGAATCTGCCCCGGTTTGAATTTCGGGTTGGC
AAATGTTGAGGTCCCATTGGCACAGCTTCTTTACCACTTCGACTGGAAGTTGGCGGAAGGAATGAAGCCTT
CCGATATGGACATGTCTGAGGCAGAAGGCCTTACCGGAATAAGAAAGAACAATCTTCTACTCGTTCCCACAC
CCTATCATCCTTCCTCCTCATGA

>Seq4 [organism=Mentha canadensis] isopiperitenol dehydrogenase
ATGGCAATCGTGAAGAAGCTCGCAGGCAAGGTAGCCATCGTAACCGGCGGCGCCAGCGGCATCGGCGAGG
TCACCGCCCGCCTCTTCGCCGAGCGCGGCGCACGCGCGGTGGTGATCGCCGACATGCAGCCCGAGAAGGG
CGGTACCGTGGCGGAATCCATCGGTGGCCGGCGGTGCAGCTACGTCCACTGCGACATCACCGACGAGGAA
CAGGTCAGGTCCGTCGTGGATTGGACCGCCGCCACCTACGGCGGCGTCGACGTGATGTTCTGCAACGCCGG
CACCGCCAGCGCCACCGCTCAGACCGTCCTGGACCTGGACCTGGCGCAGTTCGACCGCGTCATGCGCGTCA
ACGCCCGCGGCACGGCGGCGTGCGTGAAGCAGGCGGCGCGGAAGATGGTGGAGCTGGGGAGGGGAGG
CGCTATCATCTGCACCGCAAGCGCGACGGCGAACCACGCCGGTCCCAACTTGACGGACTACATCATGTCGA
AGCGCGGGGTGCTGGGGCTGGTGCGGTCGGCGAGTTTGCAGCTCGGGGTGCACGGGATTAGGGTTAACA
GCGTGTCGCCGACGGCGCTGGCCACGCCGCTCACCGCGACGATCGGGCTCCGGACGGCCGCCGATGTGGA
GAGCTTCTATGGGCAGGTCACGAGCTTGAAAGGGGTGGCGATCACGGCGGAGCACGTGGCCGAGGCGGT
GGCGTTTCTGGCTTCGGATGAGGCGGCGTTCGTCACCGGCCATGATTTGGCTGTGGATGGTGGATTGCAGT
GTTTACCATTCGTGGCCGTGGCCAAGTGA

>Seq5 [organism=Mentha canadensis] isopiperitenone reductase
ATGGCAAATGTACAGAGGTATGCATTGGTGACGGGAGCAAATAAAGGAATCGGGTTCGAAATCTGCAGGCA
GTTAGCAGAAAAAGGAATCATTGTGATTTTAACGGCAAGAAATGAAAAGAGAGGCATCGAAGCTCACCAA
AGGCTGCTTAAGGAATTAAACATTTCTAAAAATCATTTGGTTTTTCATCAACTCGATGTTACCGATCCTGCTAG
CATCGCCGCTGTTGCCGTCTTCATCAAATCTACATTCGGAAAACTCGATATTCTGGTGAATAATGCCGGAGTTA
GCGGAGTAGAGATGGTTGGAGATGTTTCCGTGTTCAACGAGTATATTGAGGCTGACTTCAACGCCCTTCAA
GCACTCGAAGCTGGTGCAAAGGAAGAGCCGCCATTTAAGCCCAAAGCAAATGGAGAAATGATCGAAAAAT
TCGAAGGAGCCAAAGATTGCGTCGAAACAAACTACTATGGTCCAAAGAGACTAACACAAGCCCTCATTCCTC
TCTTACAACTATCTCCTTCGCCGAGAATCGTCAACGTCTCCTCCTCCTTCGGGAGTTTACTGCTTCTGTGGAAC
GAATGGGCAAAGGGAGTGCTGGGGGACGAGGATAGGCTGACGGAAGAGAGAGTGGACGAAGTAGTGGA
GGTTTTTCTCAAGGATATAAAAGATGGTAAGCTCGAAGAAAATCAATGGCCTCCACATTTTGCGGCGGAAAG
AGTCTCGAAGGCGGCGCTGAACGCGTACACTAAGATCGCGGCGAAGAAGTACCCGAGTTTCCGCATAAATG
CAATATGCCCCGGTTATGCGAAAACCGACATTACCTTCCATGCGGGACCTTTGAGTGTGTCGGAAGCTGCCC
AGGTTCCGGTGAAGCTGGCTCTGCTGCCTGATGGTGGGCCCTCCGGGTGCTTCCTCCCGCGAGACAAAGCT

TTGGCTTTGTATTAA

>Seq6 [organism=Mentha canadensis] pulegone reductase
ATGGAAGTGGACATGGTGATGAACAAGCAAATTGTACTCAACAACTACGTCAACGGTTCTTTAAAAGAATCC
GACTTGTCGTTGAGAACTTCCACGATCTGCATGGAGATCCCACATGGCTGCAACGGTGCCGTTTTGGTCAAG
AACTTGTACTTGTCCGTCAATCCTTATCTCATTCTTCGCATGGGAAAACTCGATATCCCACAGTTTGATTCCATC
CTTCCTGGCTCTACTATTGTTAGCTATGGAGTGTCAAAAGTATTGGATTCGACGCATCCGAGTTACGAGAAAG
GCGAACTGATTTGGGGGTCACAAGCTGGATGGGAGGAATATACCCTTATCCAAAATCCATATAATTTGTTTAA
AATCCAAGACAAAGATGTGCCTTTATCCTACTATGTTGGAATTCTAGGAATGCCTGGGATGACAGCATATGCA
GGGTTTTTTGAGATTTGCTCTCCGAAAAAAGGCGAAACTGTGTTTGTAACGGCTGCAGCAGGATCTGTGGG
CCAGCTTGTTGGTCAGTTTGCAAAGATGTTTGGGTGCTATGTTGTTGGAAGTGCAGGGAGCAAAGAGAAG
GTTGATCTTTTGAAGAACAAATTTGGGCTCGATGATGCATTTAATTATAAAGAAGAGAGTGATTATGATACTG
CTTTGAAGAGGCACTTCCCCGAAGGAATTGATATATACTTCGATAATGTTGGAGGGAAGATGCTTGAAGCTG
TGATCAACAACATGAGAGTCCACGGCCGCATCGCGGTATGTGGGATGGTCTCCCAGTATAGCCTGAAGCAGC
CCGAAGGCGTCCACAACTTGCTTAAGCTCGTCCCAAAACAAATTCGTATGCAAGGGTTTGTCGTTGTTGATT
ACTATCATCTCTACCCAAAGTTCCTTGAAATGGTTCTGCCTTGCATCAAGGGAGGAAAAGTGATATACGTCGA
AGACATATCTGAAGGCCTTGAGAGTGCGCCTAGTGCTCTCTTGGGGGTGTATGTCGGTCGTAACATTGGCAA
TCAGGTTGTTGCCGTTTCTCGCGAGTAA

>Seq7 [organism=Mentha canadensis] menthone:menthol reductase
ATGGCAGATACTTTTACACAGAGGTATGCTTTGATTACCGGTGCAAACAAAGGAATCGGGTTCGAGATATGC
AGGCAGTTAGCTTCGAAAGGAATGAAGGTGATTTTAGCTTCAAGAAACGAGAAGAGAGGCATCGAAGCTC
GAGAAAGGCTACTTAAGGAATCGAGATCAATTTCTGATGACGATGTTGTTTTTCATCAACTTGATGTTGTTGA
CCCTGCTAGCGCTGTTGCTGTTGCTCACTTCATCGAAACCAAATTCGGGAGGCTTGATATTCTGGTGAATAAT
GCGGGATTTACTGGAGTAGCGATAGAGGGAGATATTTCAGTGTATCAAGAGTGTATTGAGGCAAACATTATT
GCAGCTCAGAGTGGACAGGCACGTCCATTCCATCCCAAATCAAATGGTAGGCTTATTGAAACATTGGAGGG
TTCAAAAGAGTGTATAGAAACAAACTACTATGGCACAAAAAGAATAACAGAAACCCTAATTCCTCTCCTGCA
AAAATCTGATTCGCCAACAATTGTCAATGTCTCATCCACCTTCTCCACTTTACTGCTCCAGCCTAATGAATGGG
CAAAAGGAGTATTTAGCAGTGAAGACACCAGTCTGAATGAAGAGAAGGTGGAGGAAGTTTTGCATGAATT
TATGAAGGATTTCATCGATGGTAAACTGCAACAAAACCACTGGCCTCCTAACCTTGCAGCCTACAAAGTGTC
GAAAGCTGCTGTTAATGCATATACTCGGATCATAGCGCGAAAGTACCCGAGTTTCTGCATAAATTCAGTGTGT
CCTGGTTTTGTTAGAACAGATATTTGCTACAATCTTGGAGTACTAAGTGAAGCTGAAGGTGCTGAAGCTCCG
GTGAAGCTGGCTTTGTTGCCCAATGGCGGGCCCTCGGGCTCCTTCTTCTCTCGAGAGGAAGCCTTGTCCTT
GTACTAA

>Seq8 [organism=Mentha canadensis] menthone:neomenthol reductase
ATGGGAGATGAAGAAGTCGTCCACCACGCTACAACAAAGAGGTATGCACTGGTTACCGGTGCGAACAGAG
GAATCGGGTTCGAAATCTGCAGGCAGTTAGCTTCCAAAGGAATCGTGGTGATTTTAGCTTCAAGAAACGAG
AAGAGAGGTATCGAAGCTCGAGAAAGGCTGATTAAGGAATTGGGATCAGAGTTTGGAAATTATGTGATTTT
TCATCAACTCGATGTTGCTGATCCTGCTAGCCTTGCCGCTCTTGTCGACTTCATCAAAACCAAATTTGGAAGC
CTTGATATTCTGGTGAATAATGCAGGAATCAACGGAGTAGAGGTGGAGGGAGATGTTTCGGTTTATACAGA
GTATGTTGAGGCAGAATTGAAGACGATGCTTGAAGCTGGTCATGGTGGAGTACAGGGAGAGGCATTTCATC
CTCAAGGAAATGGAAGGTTTGTTGAGACATTGGAGAGTGCAAAAGAGTGCATAGAAACAAACTACTATGGC
GCAAAAAGAATAACACAAGCCCTCATTCCTCTTTTGCAACTCTCTCATTCTCCAAGAATTGTCAATGTCTCCTC

TTCATTAGGGAGTTTAGTGCTTCAGCCTAATGAATGGGCAAAAGGAGTGTTTAGCAGTGAAGACAACAGCC
TCACAGAGGAGAAAGTGGAGGAAGTTTTGCATGAATTTCTCAAGGATTTCAAAGAGGCTAAACTGCAAGA
AAACCACTGGCCTCATCACTTTGCAGCCTACAAAGTGTCGAAAGCAGCTATGAACGCGTATACTCGGATTATA
GCCAAAAAATACCCGAGTTTCTGCATAAATTCCGTGTGTCCTGGTTTTACTAGAACTGAGATTTCCTACGGTC
TTGGGCAATTTAGTGTAGCTGAATCTACTGAAGCTCTGGTGAAGCTGGCTTTGCTGCCCGACGGAGGGCCG
TCCGGATGCTTCTTCACTCGAGACGAAGCGTTCTGCTTGTATTAA
