# Supplementary material for: Transcriptome Analysis of Light-Regulated Monoterpenes Biosynthesis in Leaves of Mentha canadensis L
Source: Plants (Basel). 2021 May 7;10(5):930. doi: 10.3390/plants10050930 (PMC8148558; doi:10.3390/plants10050930)
Supplement: Supplementary file 1 [file plants-10-00930-s001.zip › supplementary Table S1.pdf]

Supplemental Table S1. Summary of Illumina HiSeq4000 transcriptome sequencing and de novo assembly results

**Samples**	**CK-1**	**CK2**	**CK3**	**Dark-1**	**Dark2**	**Dark3**	**L1**	**L2**	**L3**
Before Trimming									
Number of raw reads	48817260	7239548	51753350	73818268	67944906	54548164	58076350	66576074	81571672
Number of clean reads	48726878 (99.81%)	72260904 (99.81%)	51668206 (99.84%)	73668546 (99.80%)	67815002 (99.81%)	54425488 (99.78%)	57945996 (99.78%)	66442168 (99.80%)	81427428 (99.82%)
GC content (%)	51.49	51.42	51.56	51.56	51.16	51.23	51.10	50.55	51.07
Q30 percentage (%)	93.32	92.66	93.25	93.32	93.49	92.86	92.55	93.47	93.41
Clean base (bp)	7270597723	10772650770	7713047302	10993227917	10128476398	8133079243	8620037624	9930709751	12138453272
After Assembly									
Total assembled bases (bp)	72145391								
Total unigene numbers	95579								
GC percentage (%)	43.90								
N50 number	1260								
Average length (bp)	754								
