# Supplementary material for: Transcriptome Analysis of Light-Regulated Monoterpenes Biosynthesis in Leaves of Mentha canadensis L
Source: Plants (Basel). 2021 May 7;10(5):930. doi: 10.3390/plants10050930 (PMC8148558; doi:10.3390/plants10050930)
Supplement: Supplementary file 1 [file plants-10-00930-s001.zip › supplementary Table S2.pdf]

Supplemental Table S2. Functional annotations of unigene Statistics from transcriptoms of *Mentha canadensis*

Annotation database	Number of unigenes	Percentage (%)
Nr	54084	56.58
Swissprot	32399	33.89
KOG	26218	27.43
KEGG	46665	23.82
Annotated in at least one database	61820	48.82
Total unigenes	95579	100
