# Supplementary material for: Transcriptome Analysis of Light-Regulated Monoterpenes Biosynthesis in Leaves of Mentha canadensis L
Source: Plants (Basel). 2021 May 7;10(5):930. doi: 10.3390/plants10050930 (PMC8148558; doi:10.3390/plants10050930)
Supplement: Supplementary file 1 [file plants-10-00930-s001.zip › Supplementary Table S3.pdf]

Supplementary Table S3. Sequences of primers used for qRT-PCR

**Unigenes**	**Forward primers**	**Reverse primers**
Unigene0016246 (COP1)	5’- ATGCGTTTCTGACTTCGTG -3’	5’- AAGGAGTGGCAGTTTTAGC -3’
Unigene0003571 (PHOT)	5’- TTGATTGGTGGGCTCTTGG -3’	5’- GCCTTGCTGGGAGTGTTAC -3’
Unigene0009588 (WRKY40)	5’-GATCTAATAATGCCCTCCCT-3’	5’-TCACGCTCTTCACCACCT-3’
Unigene0055177 (ERF1)	5’- TGGCGGACAATTAGTGGTG -3’	5’- AAGCAAGTGGGAAAATGGG -3’
Unigene0018692(MsMYB)	5’- GTATGACGGAGGACTTGAAC -3’	5’- TCCCAAATCCACGAAAGCG -3’
Unigene0003114 (MYC1)	5’- GAAGAGGGGAAGGAAGCC -3’	5’- TGCGAGATACGTTGGGGA -3’
Unigene0000987 (CPRF3)	5’- GGGTGACATCATGAAGGC-3’	5’- CAAGATACTCTGCTCTGCG-3’
Unigene0003421 (RAV1)	5’- CTCCAAACAGCCTCACCAT -3’	5’- CGCCGGAAAAGTTTGGAGA -3’
Unigene0045757(GPPS-l)	5’-TACATCCACCACCACAAGACGG-3’	5’-TTCCTGAGCCTCGCCACCT-3’
Unigene0038587 (GPPS-s)	5’-CGTCCACGAACACCTCCCT-3’	5’-CCGGCCAGCAACTCAAAC-3’
Unigene0018537 (LS)	5’-CGACCAACTTCCCGATTA-3’	5’-AACCACCGTGCCTCTACC-3’
Unigene0052571 (L3OH)	5’-CCCGAAAGGTTTGACGATGT-3’	5’-CAGATTCTTCTTCCCGCTCC-3’
Unigene0033747 (iPD)	5’-GCGACATCACCGACGAGGAA-3’	5’-GCCCGCGTTGCAGAACAT-3’
Unigene0033916 (iPR)	5’-GTTGGAGATGTTTCCGTGTT-3’	5’-TGGACCATAGTAGTTTGTTTCG-3’
Unigene0030907 (MFS)	5’-AGAGGGTCCAGTCGTTCCG-3’	5’-ACTACGCCGTTCGTCAGC-3’
Unigene0047772 (PR)	5’-GCGTCCACAACTTGCTTA-3’	5’-TACGACCGACATACACCC-3’
Unigene0041050 (MR)	5’-ACTGGAGTAGCGATAGAGGG-3’	5’-GATTTGGGATGGAATGGAC-3’
Actin	5’-CCAGGAATTGCTGATAGGATGAG-3’	5’-GCGCCACCACCTTAATCTTC-3’
