# Supplementary figures and images for: Transcriptome Analysis of Light-Regulated Monoterpenes Biosynthesis in Leaves of Mentha canadensis L
Source: Plants (Basel). 2021 May 7;10(5):930. doi: 10.3390/plants10050930 (PMC8148558; doi:10.3390/plants10050930)

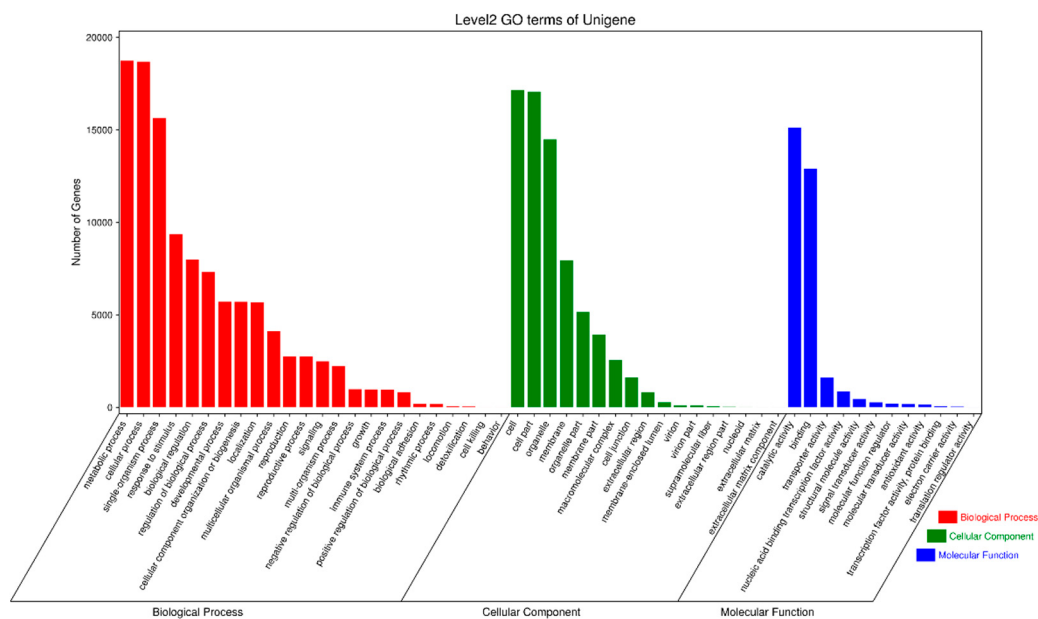

Supplementary Figure S1 GO functional classification of assembled unigenes

Supplement: Supplementary file 1 [file plants-10-00930-s001.zip › Supplementary Figure S1.pdf]

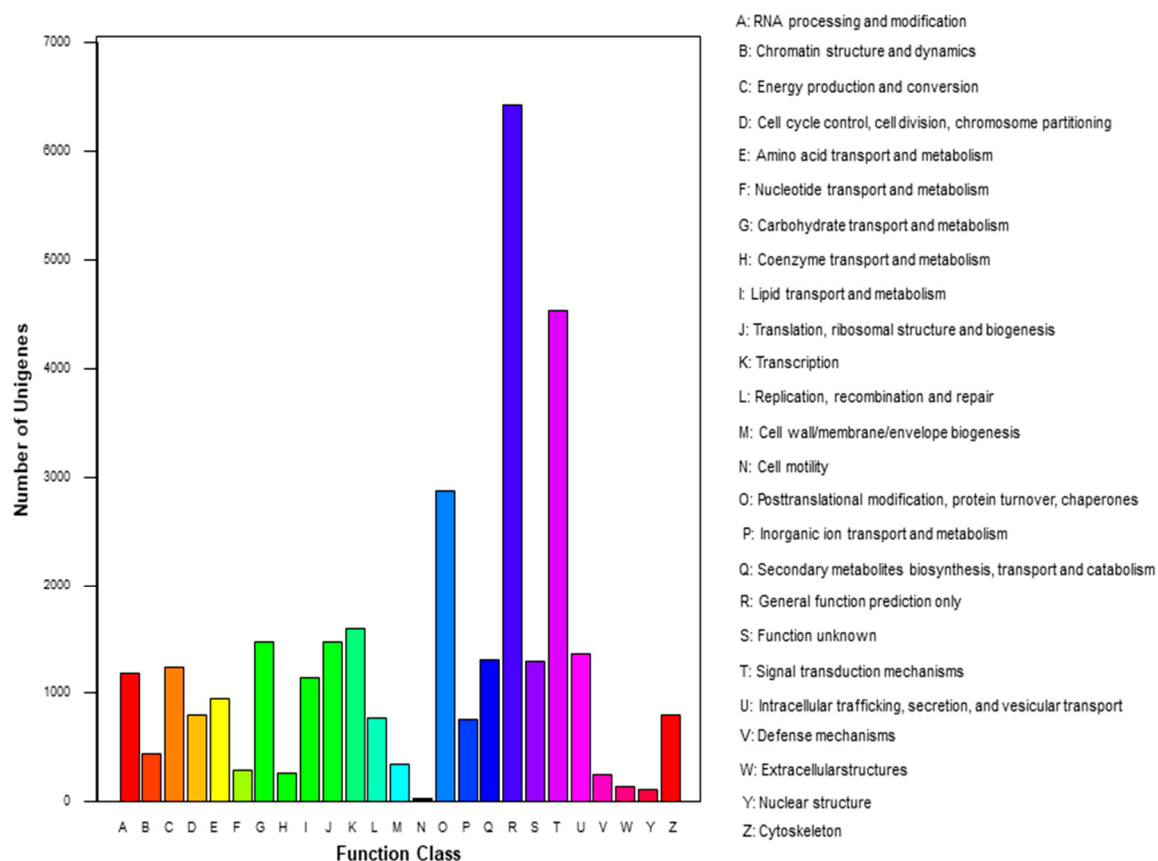

Supplementary Figure S2 KOG functional classification of assembled unigenes

Supplement: Supplementary file 1 [file plants-10-00930-s001.zip › Supplementary Figure S2.pdf]

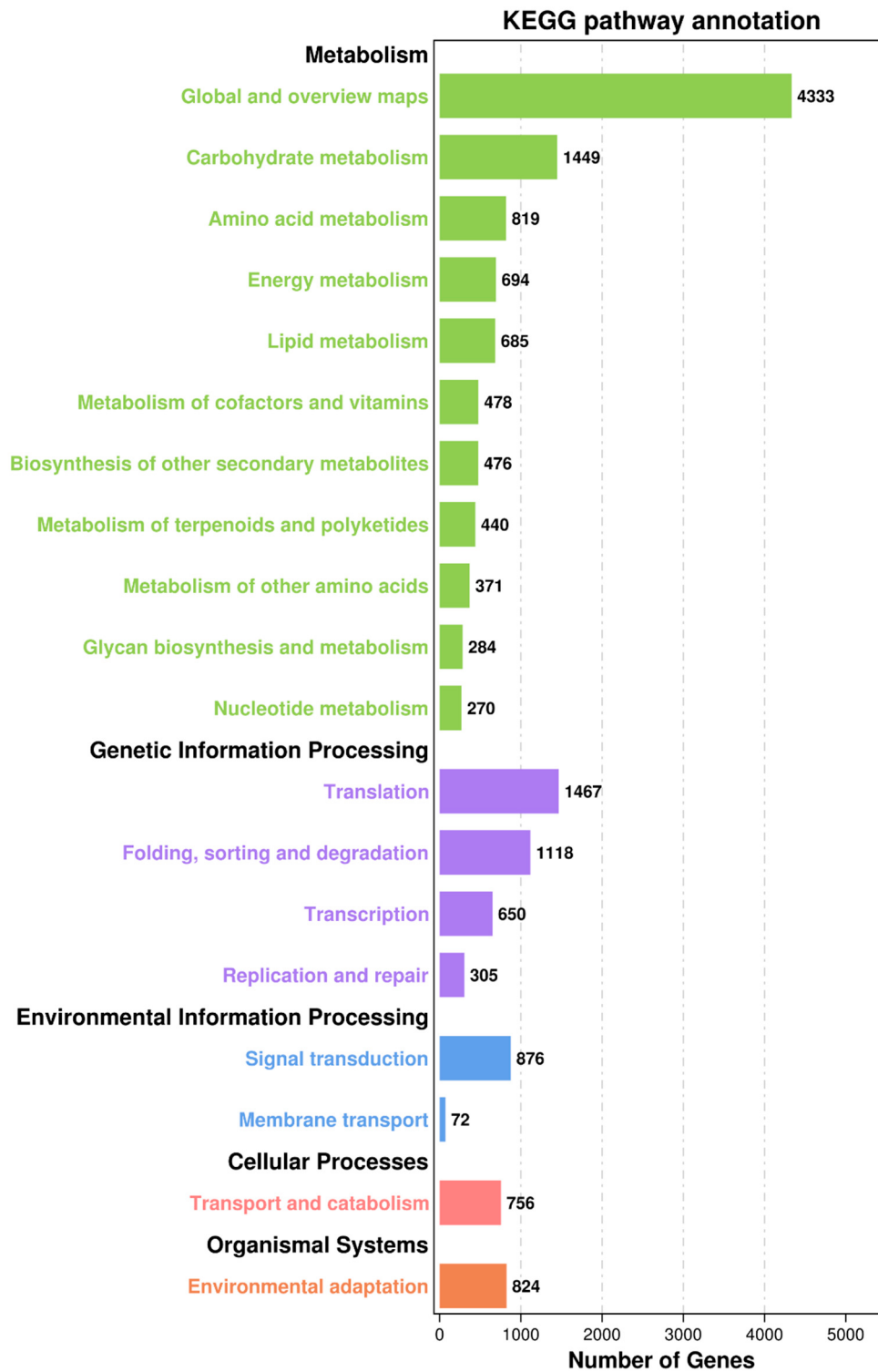

**Supplementary Figure S3 KEGG pathway annotation of assembled unigenes.**

Supplement: Supplementary file 1 [file plants-10-00930-s001.zip › Supplementary Figure S3.pdf]
