# Supplementary material for: Transcriptome Analysis of Light-Regulated Monoterpenes Biosynthesis in Leaves of Mentha canadensis L
Source: Plants (Basel). 2021 May 7;10(5):930. doi: 10.3390/plants10050930 (PMC8148558; doi:10.3390/plants10050930)
Supplement: Supplementary file 1 [file Supplementary Figure S5 and Figure S6.pdf › Supplementary Figure S5 and Figure S6.pdf]

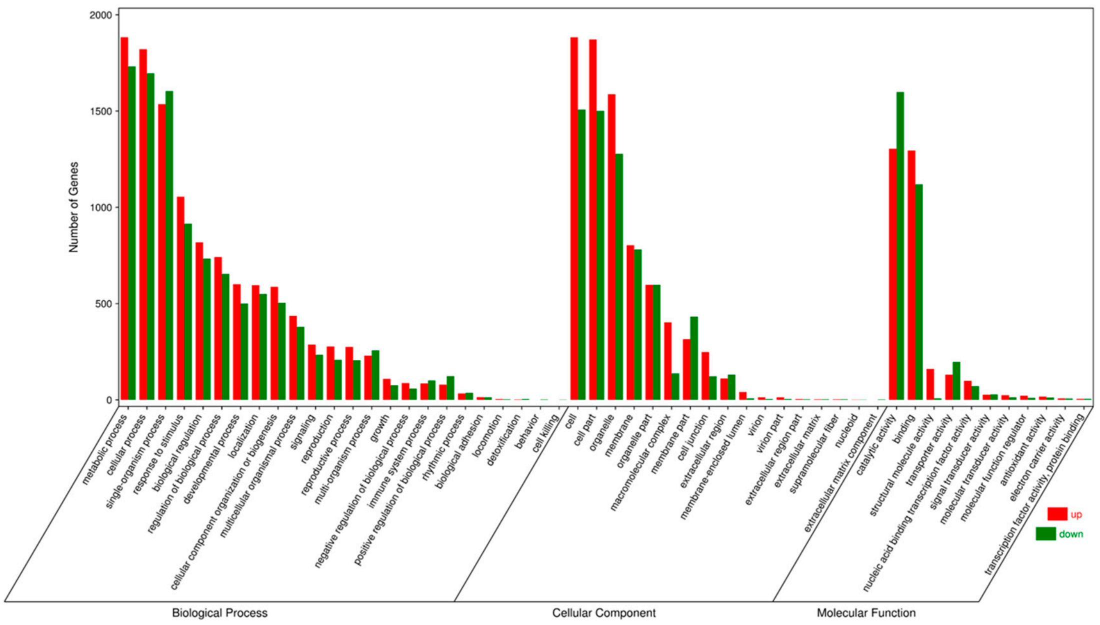

Supplementary Figure S5 GO terms of DEGs among control light condition vs darkness

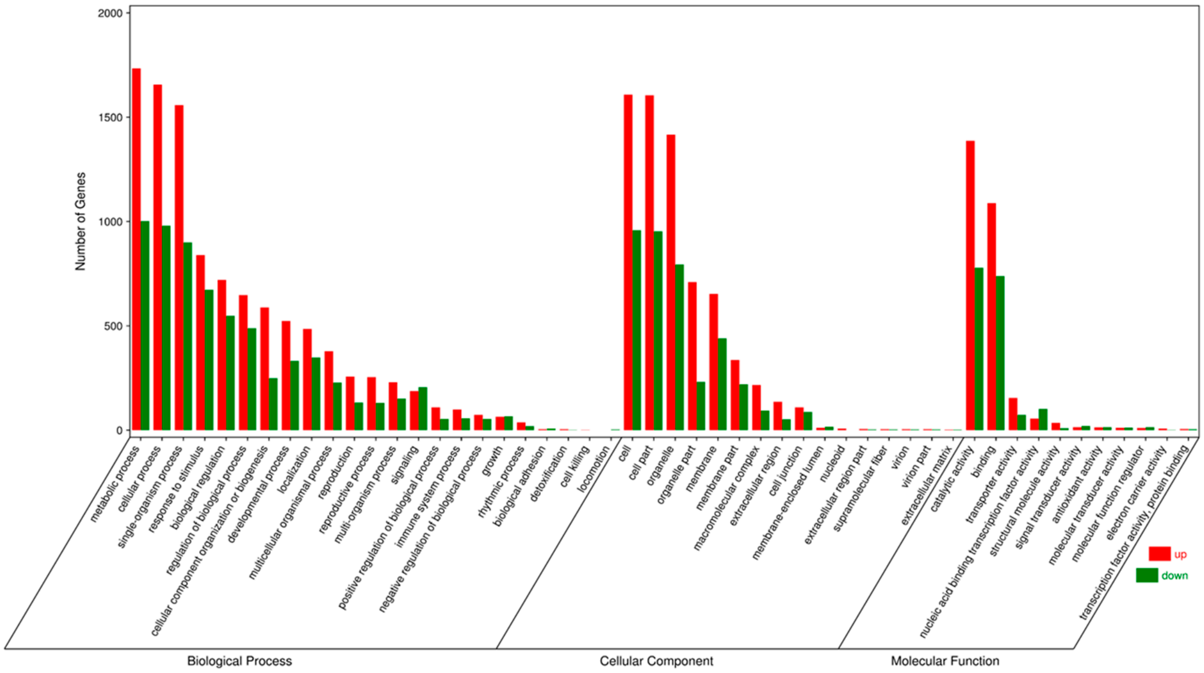

Supplementary Figure S6 GO terms of DEGs among darkness condition vs exposure to light
